# Cross-Regulation between the *phz1* and *phz2* Operons Maintain a Balanced Level of Phenazine Biosynthesis in *Pseudomonas aeruginosa* PAO1

**DOI:** 10.1371/journal.pone.0144447

**Published:** 2016-01-06

**Authors:** Qinna Cui, Huinan Lv, Zhuangzhuang Qi, Bei Jiang, Bo Xiao, Linde Liu, Yihe Ge, Xiaomei Hu

**Affiliations:** 1 Department of Applied and Environmental Microbiology, School of Biological Sciences, Ludong University, Yantai, China; 2 Department of Microbiology, College of Basic Medical Sciences, Third Military Medical University, Chongqing, China; University of North Dakota, UNITED STATES

## Abstract

Gene duplication often provides selective advantages for the survival of microorganisms in adapting to varying environmental conditions. *P*. *aeruginosa* PAO1 possesses two seven-gene operons [*phz1* (*phzA1B1C1D1E1F1G1*) and *phz2* (*phzA2B2C2D2E2F2G2*)] that are involved in the biosynthesis of phenazine-1-carboxylic acid and its derivatives. Although the two operons are highly homologous and their functions are well known, it is unclear how the two *phz* operons coordinate their expressions to maintain the phenazine biosynthesis. By constructing single and double deletion mutants of the two *phz* operons, we found that the *phz1*-deletion mutant produced the same or less amount of phenazine-1-carboxylic acid and pyocyanin in GA medium than the *phz2-*knockout mutant while the *phz1*-*phz2* double knockout mutant did not produce any phenazines. By generating *phzA1* and *phzA2* translational and transcriptional fusions with a truncated *lacZ* reporter, we found that the expression of the *phz1* operon increased significantly at the post-transcriptional level and did not alter at the transcriptional level in the absence of the *phz2* operon. Surprisingly, the expression the *phz2* operon increased significantly at the post-transcriptional level and only moderately at the transcriptional level in the absence of the *phz1* operon. Our findings suggested that a complex cross-regulation existed between the *phz1* and *phz2* operons. By mediating the upregulation of one *phz* operon expression while the other was deleted, this crosstalk would maintain the homeostatic balance of phenazine biosynthesis in *P*. *aeruginosa* PAO1.

## Introduction

Phenazines are an array of secondary metabolites that are biosynthesized and secreted by fluorescent pseudomonad. Many studies have reported that phenazines play a major role in microbial competitiveness [1,2], suppression of soil-borne plant fungal pathogens [3–6], and affect their pathogenicity in human or animal hosts [7,8].

Of all the phenazine-producing microorganisms, the major opportunistic pathogen *Pseudomonas aeruginosa* is the most widely studied phenazine-producing bacterium. *P*. *aeruginosa* has been identified as a common pathogen in animals, insects, nematodes, and plants [8–11]. In the human host, *P*. *aeruginosa* causes severe and chronic infections in immunocompromised, burned, and injured patients [12]. Additionally, *P*. *aeruginosa* is the most commonly found pathogen associated with cystic fibrosis (CF) in patients’ lung and is responsible for progressive lung tissue destruction leading to respiratory failure [13,14].

*P*. *aeruginosa* produces a common precursor phenazine-1-carboxylic acid (PCA) that is biosynthesized into its main derivatives pyocyanin (PYO), 1-hydrophenazine (1-OH-PHZ), and phenazine-1-carboxamide (PCN) [1, 15–17]. It was reported that at least 90% of *P*. *aeruginosa* isolates could produce PYO [17,18]. Moreover, PYO was detected at high concentrations in the sputum of cystic fibrosis patients, suggesting that phenazine compounds could act as virulence factors and play a crucial role in host-pathogen interactions [19,20]. This hypothesis is supported by several studies on the pathophysiological effects of PYO and other phenazine derivatives found in the airways of individuals infected with *P*. *aeruginosa*. For example, it was proposed that PCA and PYO were responsible for increasing oxidant production, neutrophil chemokine IL-8 and leukotriene B_4_ release, and the expression of intercellular adhesion molecule-1 (ICAM-1) by human airway epithelial cells [21–23]. PYO could also inhibit the cytokine-dependent expression of RANTES, and monocyte chemoattractant protein-1 (MCP-1) [23–25]. Moreover, PYO was recently shown to cause airway goblet cell hyperplasia and metaplasia and mucus hypersecretion in airway epithelial cells [26].

Two copies of the seven-gene operon *phz1* (*phzA1B1C1D1E1F1G1*) and *phz2* (*phzA2B2C2D2E2F2G2*) are known to be responsible for the biosynthesis of PCA in *P*. *aeruginosa* and *Streptomyces cinnamonensis* [17,27,28]. In these strains, the *phz1* and *phz2* operons share 99% identity and possess similar flanking genes respectively. Gene duplication is often found in many microorganisms and is thought to provide several selective advantages when the bacteria encounter various environments [29]. For example, the maintenance of duplicate genes may be favored when spatial or temporal differences in expression enable tissue-specific variation or survival under varying environmental conditions [30,31]. In *P*. *aeruginosa* PA14, the two *phz* operons showed environment-dependent expression and played differential roles in its pathogenicity [32].

In the PAO1 strain, the *phz1* is located at positions 4,713,795 to 4,720,062 bp in the genome, while the *phz2* is located approximately 2.6 Mb from *phz1* at positions 2,070,685 to 2,076,985 bp. Although the two *phz* operon exhibit 98.3% identity at the DNA level, their promoter regions are quite different, indicating that *phz1* and *phz2* may be modulated via different regulation mechanisms [17]. Both the PQS and *rhl* systems positively regulate *phz1* expression [29,30], while the orphan LuxR-type quorum sensing regulator QscR negatively regulates *phz1* and *phz2* expression [12,31]. Although both *phz1* and *phz2* contribute to the production of phenazines, *phz1* expression has been proposed to account for the majority of phenazines biosynthesis based on regulation analysis [33,34]. However, it is now known if the *phz1* and *phz2* operons cross-regulate each other during phenazine biosynthesis. In this study, we first generated mutants lacking the *phz1* and/or *phz2* operons and evaluated phenazine biosynthesis in the PAO1 strain. Because PCA and PYO of phenazines produced by the *phz1* or *phz2* operons differed from those reported in the PA14 strain during growth in liquid batch cultures [32], we employed promoterless *lacZ* fusions constructed on a plasmid and the chromosome to examine the expression of the *phz1* and *phz2* operons at the transcriptional and post-transcriptional level. Our results indicated that a cross talk could exist between the *phz1* and *phz2* operons in thePAO1 strain. This cross-regulation between the two *phz* operons may function to balance phenazine biosynthesis homeostatically.

## Materials and Methods

### Bacterial strains, plasmids, primers and culture conditions

All bacterial strains and the primary plasmids and primers used in this study are shown in Tables 1 and 2, respectively. Cultures of *Escherichia coli* were routinely grown in Luria-Bertani (LB) medium at 37°C [35]. *P*. *aeruginosa* PAO1 and its derivatives were routinely grown at 37°C in LB broth with shaking at 180 rpm, or on LB agar sometimes amended with sucrose (10%) for screening double-cross mutants, or in glycerol-alanine supplemented (GA) medium for the PCA and PYO assays [36]. The antibiotics applied to the medium included spectinomycin (Sp, 100 μg/ml), tetracycline (Tc, 125 μg/ml), kanamycin (Km, 300 μg/ml) or gentamycin (Gm, 40 μg/ml) in the experiments with the PAO1 strain and its derivatives and ampicillin (Ap, 50 μg/ml), Tc (25 μg/ml), Km (50 μg/ml) or Gm (20 μg/ml) in the experiments with *E*. *coli*.

**Table 1:** Bacterial strains and plasmids.

Strain/plasmid	Relevant characteristics	Source/reference
Strains		
*E*. *coli*		
DH5α	Φ80 *lacZ*ΔM15 Δ (*lacZYA-argF*) U169 *hsdR17 recA1endA1 thi*-1	Lab collection
SM10	F^-^ *thi-1 thr-1 leuB6 recA tonA21 lacY1 supE44*(Mu_C_^+^) λ^-^ Km^r^	Lab collection
*P*. *aeruginosa*		
PAO1	Phenazine-1-carboxylic acid and its derivatives producer, Wild type, Ap^r^Sp^r^	Lab collection
Δ*phz1*	*phz1* locus deleted and inserted with *aacC1*, Sp^r^Gm^r^	This study
Δ*phz2*	*phz2* locus deleted and inserted with *aph*, Sp^r^Km^r^	This study
Δ*phz1phz2*	*phz1* deleted and inserted with *aacC1*, *phz2* deleted and inserted with *aph*, simultaneously, Gm^r^Km^r^	This study
Δ*phzA2Z*	the partial *phzA2B2* deleted and chromosomally fused with the truncated *lacZ* in frame, Sp^r^	This study
Δ*phz1phzA2Z*	*phz1*deleted and inserted with *aacC1* in the mutant Δ*phzA2Z*, Sp^r^Gm^r^	This study
Δ*phzA1Z*	the partial *phzA1B1* deleted and chromosomally fused with the truncated *lacZ* in frame, Sp^r^	This study
Δ*phzA1Zphz2*	*phz2* deleted and inserted with *aph* in the mutant Δ*phzA1Z*, Sp^r^Km^r^	This study
Plasmids		
pBluescript II SK	Clone vector, ColE, Ap^r^	Stratagene
pGEM-T	T-vector, ColE, Ap^r^	Promega
pEX18Tc	Gene replacement vector with MCS from pUC18, oriT^+^ sacB^+^, Tc^r^	[39]
pEXZ1	pEX18Tc containing a 2.0-kb *phz1*-flanking PCR fragment, Tc^r^	This study
pEXZ1G	A 2.0-kb *phz1*-flanking PCR fragment inserted with *aacC1* in pEX18Tc, Tc^r^Gm^r^	This study
pEXZ1Z	A 2.4-kb *phzA1B1*-deleted PCR fragment cloned in pEX18Tc, Tc^r^	This study
pEXZ1Zlac	A 2.4-kb *phzA1B1*-deleted PCR fragment fused in frame with the truncated *lacZ* in pEX18Tc, Tc^r^	This study
pEXZ2	pEX18Tc containing a 3.0-kb *phz2*-flanking PCR fragment, Tc^r^	This study
pEXZ2K	A 3.0-kb *phz1*-flanking PCR fragment inserted with *aph* in pEX18Tc, Tc^r^Km^r^	This study
pEXZ2Z	A 2.5-kb *phzA2B2*-deleted PCR fragment cloned in pEX18Tc, Tc^r^	This study
pEXZ2Zlac	A 2.5-kb *phzA2B2*-deleted PCR fragment fused in frame with the truncated *lacZ* in pEX18Tc, Tc^r^	This study
pME10Z1	pME6010 containing a 6.9-kb *phz1* cluster, Tc^r^	This study
pME10Z2	pME6010 containing a 6.8-kb *phz2* cluster, Tc^r^	This study
pME15Z1	A 0.9-kb *phz1* upstream fragment and a translational *phzA1′-′ lacZ* fusion with first 8 *phzA1* codons in pME6015, Tc^r^	This study
pME15Z2	A 0.9-kb *phz2* upstream fragment and a translational *phzA2′-′ lacZ* fusion with first 8 *phzA2* codons in pME6015, Tc^r^	This study
pME22Z1	pME6522 carrying a 902-bp upstream region of *phz1* (from -902 to +1) and transcriptional fusion *phz1-lacZ*, Tc^r^	This study
pME22Z2	pME6522 carrying a 517-bp upstream region of *phz2* (from -517 to +1) and transcriptional fusion *phz2 -lacZ*, Tc^r^	This study
pME6010	Low capy vector in *Pseudomonas* sp., Tc^r^	[43]
pME6015	pVS1-p15A shuttle vector for translational *lacZ* fusion, Tc^r^	[43]
pME6522	pVS1-p15A shuttle vector for transcriptional *lacZ* fusion and promoter probing, Tc^r^	[44]
pNM481	′*lacZ* fusion vector, Ap^r^	[45]
pNM482	′*lacZ* fusion vector, Ap^r^	[45]
pUC18-19Km	ColE, *aph*, kanamycin resistance cassette flanked with multiple restriction sites, Ap^r^Km^r^	[42]
pUCGm	ColE, *aacC*, gentamycin resistance cassette flanked with multiple restriction sites, Ap^r^Gm^r^	[40]

**Table 2:** PCR primers used in this study.

Primers	Sequences (5'→3', artificial restriction enzyme site underlined and in italics)
phz1-1F	GGA CGG CAC CTC TTG CAG CAT G
phz1-1R	AAA TTT *TCT AGA* CTT TCA GCG TCA TTC CGT G (*Xba*I)
phz1-2F	CAA TTA *TCT AGA* GCC CAT CTA ACC GCA CGC GGT C (*Xba*I)
phz1-2R	CCA GCT CGA TGC CGT CGA GGA TTG C
phz1-3F	AAA TTT *GAG CTC* CCC TGC CAA CAG GCT GG (*Sac*I)
phz1-3R	GTA TAT *AAG CTT* GCG AAG CGC CGT TGG CG (*Hin*dIII)
phz2-1F	CAT CCA TTT GTT CCA GGT GAT GCC
phz2-1R	TTA ATT *GGT ACC* TAA TGC CGA ATT GCC ATG ACC G (*Acc*65I)
phz2-2F	CAA TAT *GGT ACC* TGC AAC CGT GAC GAC ACC G (*Acc*65I)
phz2-2R	GCC CGC CCG AGA AGC TTC AAC G
phz2-3F	AAT TAA *GAG CTC* GAC ACC TGG ACG ATG TTG AGG AAG (*Sac*I)
phz2-3R	GTA TCT *AAG CTT* CGA GCA CGC CGG CCA ACG (*Hin*dIII)
phz1z-1R	GTA CAT *AGT ACT* CGA TGT CGA GGG GTG TTT CCC TG (*Sca*I)
phz1z-2F	GAT CAT *AGT ACT* TCG CGA AAA GAA TCG CGC CAC C (*Sca*I)
phz1z-2R	AGT GGG TCG AAC CGA GAT AGA C
phz1z-3R	TAA ATT *AAG CTT* GCT CGT CCT CGC GCA GCA TCG (*Hin*dIII)
phz2z-1F	CTC TCC CGA CGA CGA TGG AGC GTG C
phz2z-1R	GTA ATT *CCC GGG* TAA ACC CTT TCA ACC GTT GGT ACT C (*Sma*I)
phz2z-2F	CAA TAT *CCC GGG* TTT CGA AGA CGC CGT GGA G (*Sma*I)
phz2z-2R	CCA CTT GGT CAG CAG CCA GTC GTC C
phz2z-3F	CAT ATA *GGT ACC* GCC GTG AGG CCC ATC GGA GAG C (*Acc*65I)
phz2z-3R	GTA CTA *TCT AGA* CCG CGC TGC TCC TCG GTC ATG C (*Xba*I)
phz1-WF	GAT TAC *AAG CTT* AGC AAT CCC GCA TAC CCT GTC (*Hin*dIII)
phz1-WR	ATA ATT *GGT ACC* GCG ATG AAA CGT CGG CGC AG (*Kpn*I)
phz2-WF	GAA TAA *GAG CTC* CTG TTG TCC GGC ACG CTA GTG (*Sac*I)
phz2-WR	GTA ATT *GAG CTC* CGA GTC CGC GCA GGA CGC ATG (*Sac*I)
phz1-LF	CTA TTA *GAA TTC* GTC GAT CCC GCT CTC GATC (*Eco*RI)
phz1-LR	GTA AAT *CTG CAG* TTC CCT GTA CCG CTG AC (*Pst*I)
phz2-LF	GTT ATA *GAA TTC* CAC GGC ATC CGT CAC (*Eco*RI)
phz2-LR	CTT AAT *GGA TCC* CAA CCG TTG GTA CTC (*Bam*HI)
phz1-CF	CAA TTA *GAA TTC* GCC GGA ACC GCC ACC GAC (*Eco*RI)
phz1-CR	GTA TTA *CTG CAG* ATT GCA TAA AAC ACA GAA CGC TC (*Pst*I)
phz2-CF	GAA TAT *GAA TTC* GGC GAC CTG CTG GCG CC (*Eco*RI)
phz2-CR	GTT ATA *CTG CAG* ACA AAC TTA TAA ACG CTT TTT TG (*Pst*I)

### DNA manipulation and cloning procedure

Small-scale plasmids were prepared from *P*. *aeruginosa* derivatives or *E*. *coli* using the alkaline lysis method or Plasmid DNA Extraction Kit (Sangon, Shanghai, China). Chromosomal DNA was isolated from *P*. *aeruginosa* with the method as described by Chen and Kuo [37] or by using the Genomic DNA Extraction Kit (Sangon, Shanghai, China). Standard DNA recombinant techniques were applied for digestion, agarose gel electrophoresis, dephosphorylation, isolation of DNA fragments from agarose gels, and ligation. *E*. *coli* or *Pseudomonas sp*. cells were transformed with plasmid DNA by CaCl_2_ treatment or electroporation, respectively [38].

Polymerase chain reactions (PCRs) were typically performed with 2.5U of thermostable DNA polymerase in a reaction mixture containing 100 ng of target DNA. A 250 μM concentration of each of the four dNTPs, 10 pmol of two primers, 5 mM MgCl_2_, and 1×buffer in a final volume of 25 μl were used for the amplification reaction. A total of 30 or 33 cycles (2 min at 94°C, 30 sec at 50 to 55°C, and 1 min 72°C) was followed by a final elongation step for 7 min at 72°C. PCR products were cloned into pGEM-T or pBluescript II SK for verification by sequencing.

### Deletion mutation of the *phz1* and/or *phz2* operons

To delete the *phz1* operon, a disruption plasmid was first created. A 1114-bp fragment covering a partial sequence of the *phzM* gene and a partial upstream region of the *phzA1* gene was amplified with primers phz1-1F and phz1-1R. A second fragment of 1170-bp which was located at the downstream region of the *phzG1* and contained a partial sequence of the *phzS* was amplified using primers phz1-2F and phz1-2R. The two PCR products were pooled, purified using the PCR purification kit (Sangon, Shanghai, China), digested with *Xba*I, repurified, and ligated. The resultant ligation served as the template, and nested PCR was performed with primers phz1-3F and phz1-3R. After double digestion with *Sac*I and *Hin*dIII, the PCR product was cloned into the suicide plasmid pEX18Tc [39], resulting in pEXZ1. A gentamycin resistance cassette (*aacC1*) was obtained via the *Xba*I-digestion of the cloning vector pUCGm [40], and cloned into the unique *Xba*I site in pEXZ1 to generate pEXZ1G.

To knock out the *phz2* operon, the same nested PCRs were performed to construct the suicide plasmid pEXZ2. Briefly, a first fragment with a length of 1987 bp containing whole *qscR* sequence and partial upstream region of *phzA2* and a second fragment of 1087 bp covering the partial downstream region of *phzG2* were amplified with two pairs of primers (phz2-1F/phz2-1R and phz2-2F/phz2-2R, respectively). After purification, digestion with *Kpn*I, and re-purification, the two PCR products were mixed and ligated. Using the ligation product as a template, an approximately 3.0-kb nested PCR product was amplified with primers phz2-3F/phz2-3R and then cloned into pEX18Tc to obtain pEXZ2. A *Kpn*I-digested kanamycin resistance cassette (*aph*) from pUC18-19Km was cloned into the unique *Kpn*I site in pEXZ2 to generate pEXZ2K [41,42].

**Fig 1:**
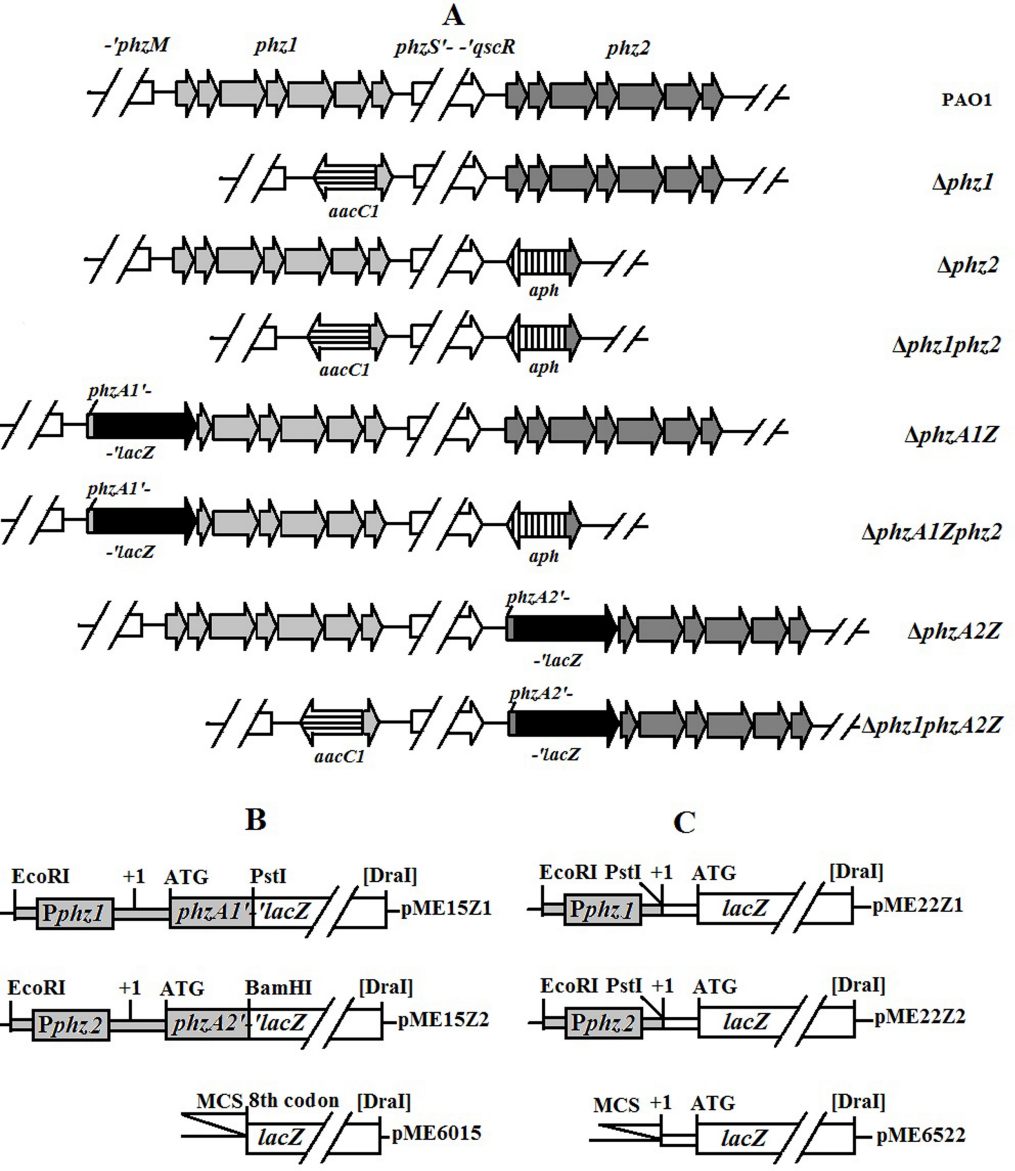
Structures of two *phz* operons in *P*. *aeruginosa* PAO1 and its derivatives and two types of plasmid fusions with the truncated *lacZ*. (A) *phz1* (light grey arrows) and *phz2* (heavy grey arrows) indicate two phenazine operons of *phzA1B1C1D1E1F1G1* and *phzA2B2C2D2E2F2G2*, respectively. *aacC1* (horizontally striped arrow) and *aph* (vertically striped arrow) indicate the gentamycin and kanamycin resistance cassettes inserted into chromosome, respectively. *lacZ* (black arrow) indicates the truncated β-galactosidase gene inserted and fused in frame with the first several codons of *phzA1* or *phzA2* and their upstream region in the chromosome. The translational plasmid fusion (B) and the transcriptional plasmid fusion (C) were generated in plasmids pME6015 and pME6522, respectively. MCS stands for the multi-cloning site.

After confirmation, the suicide plasmids pEXZ1G and pEXZ2K were mobilized from *E*. *coli* SM10 (donor strain) to *P*. *aeruginosa* PAO1 (receptor strain) by biparental mating. The *phz1*-deficient mutant (designated as Δ*phz1*) was selected on plates containing 10% sucrose and gentamycin due to its gentamycin resistance and tetracycline sensitivity. The *phz2* knockout mutant (called Δ*phz2*) was obtained with the same selection methods described above based on its kanamycin resistance and tetracycline sensitivity. Then, the double-deletion mutant Δ*phz1phz2* was constructed by mating the mutant Δ*phz1* with the pEXZ2K-harboring cells of *E*. *coli* SM10, or by mating the mutant Δ*phz2* with the pEXZ1G-bearing cells of *E*. *coli* SM10. All of the mutant constructs involved in this study are shown in Fig 1A. The insertion of the *aacC1* and *aph* resistance cassette was verified by PCR in all mutants and relevant data were available on figshare (http://dx.doi.org/10.6084/m9.figshare.1612163).

### Cloning and complementation expression of the *phz1* or *phz2* operons

To clone the *phz1* operon, a 6.9-kb fragment containing the whole *phz1* DNA region was amplified with a primer pair (phz1-WF and phz1-WR). After double digestion with *Hin*dIII and *Kpn*I, the PCR product was cloned into the low-copy shuttle vector pME6010 to obtain pME10Z1 [43]. Using the same methods, pME10Z2 covering the whole *phz2* DNA region was constructed using primers phz2-WF and phz2-WR. After sequencing, the plasmids were transformed into competent PAO1 cells or its derivatives by electroporation. The positive colonies formed on LB plates supplemented with tetracycline were confirmed by plasmid isolation and restriction enzyme digestion analysis.

### Creation of the translational fusion constructs: *phz1 '-' lacZ* and *phz2 '-' lacZ* and the transcriptional fusion constructs: *phz1-lacZ* and *phz2-lacZ*

To quantify the expression levels of the two phenazine-producing operons, the translational fusion constructs *phz1 '-' lacZ* and *phz2 '-' lacZ* were created in plasmid (Fig 1B). Briefly, a 0.9-kb DNA fragment covering the first ten codons of *phzA1* and its upstream region was amplified with a pair of primers, phz1-LF and phz1-LR. The relevant PCR product was purified, double-cleaved with *Eco*RI-*Pst*I, re-purified, and then fused in-frame with the truncated *lacZ* in plasmid pME6015 to create pME15Z1 [43]. Similarly, pME15Z2 (a translational fusion construct *phz2 '-' lacZ*) was constructed in pME6015 with a 0.9-kb fragment containing the first eight codons of *phzA2* and its upstream region amplified with a primer pair phz2-LF/phz2-LR.

To assess the two *phz* operons at the transcription level, the transcriptional fusion constructs pME22Z1 (*phz1-lacZ*) and pME22Z2 (*phz2-lacZ*) were created in plasmid (Fig 1C). Briefly, a 0.9-kb DNA fragment covering the partial downstream region of the *phzM* and the *phz1* promoter region (to transcription start site +1) were amplified with a pair of primers, phz1-CF/phz1-CR, then double digested with *Eco*RI and *Pst*I, and cloned into pME6522 to generate pME22Z1[28, 44]. Similarly, a 0.5-kb fragment of the *phz2* promoter region with the partial *qscR* gene was amplified using a primer pair phz2-CF/phz2-CR, and then cloned into the *Eco*RI-*Pst*I site in pME6522 to create pME22Z2. All of the fusions were verified by sequencing analysis prior to transformation.

### Creation of the translational *phz1* or *phz2* fusion mutants with the truncated *lacZ* in frame

To precisely reflect the expression of the *phz1* and *phz2* gene clusters in PAO1 and its derivatives, mutants in which the *phz1* or *phz2* were deleted and insertionally fused in frame with a truncated *lacZ* in their chromosome were further created. To obtain the *phz1* fusion mutant, two fragments were amplified with two pairs of primers (phz1-1F×phz1z-1R and phz1z-2F×phz1-2R) to obtain a 1432-bp fragment covering eight codons of *phzA1* and its upstream region and a 1099-bp fragment containing partial sequence of *phzB1* and *phzC1*. These two purified PCR products were cleaved with *Sca*I, re-purified and ligated. Using this ligation product as a template and a pair of primers (phz1-3F×phz1z-3R), a 2.4-kb PCR fragment was amplified, purified, double digested with *Sac*I and *Hin*dIII, and finally cloned into pEX18Tc to create pEXZ1Z. A 3.1-kb *Sma*I-*Dra*I fragment of the truncated *lacZ* from pNM482 was inserted in-frame into the *Sca*I site in pEXZ1Z to yield pEXZ1Zlac [45]. Similarly, using three pairs of primers (phz2z-1F×phz2z-1R, phz2z-2F×phz2z-2R and phz2z-3F×phz2z-3R), the suicide vector pEXZ2Zlac containing an in-frame fusion of *phzA2* with the truncated *lacZ* was constructed to obtain the *phz2* fusion mutant.

Biparental mating was performed by mobilizing the suicide vectors described above from *E*. *coli* SM10 to *P*. *aeruginosa* PAO1 or its derivatives. The potential mutants Δ*phzA1Z* or Δ*phzA2Z* that lacked tetracycline resistance were isolated following the production of visible blue colonies on LB medium plates spread with 5-bromo-4-chloro-3-indolyl-β-D-galactopyranoside (X-Gal), indicating that a double-crossover event had occurred [46]. The other set of mutants (Δ*phz1phzA2*Z and Δ*phzA1Zphz2*) were created by biparental mating using the mutant Δ*phz1* or Δ*phz2* as the receptor strain, respectively. These mutants were selected on LB medium plates containing X-Gal based on the features of blue colonies and tetracycline sensitivity (Fig 1A). All of the mutants were verified by PCR (data available on figshare).

### Quantitative assay for PCA and PYO

For PCA, the cell cultures were grown in 500-ml shaking flasks with 150 ml GA or LB broth at 37°C for 72h. 900 μl of samples were collected once every 12 hours and then acidified to pH 4.0 with HCl before adding 2.7 ml of chloroform. Chloroform extracts were clarified by centrifugation at 10,000 rpm for 5 min. Phenazine samples were diluted with chloroform appropriately, and PCA was quantified spectrophotometrically at 252 nm [47]. The equation of linear regression [concentration (μg/ml) = 2.9667×OD_252_-0.0979, R^2^ = 0.9998] was generated with a purified sample of PCA provided by Dr. Xu (Shanghai Jiaotong University, Shanghai, China) as a gift.

PYO was extracted with chloroform from cultures grown in 500-ml flasks containing 150 ml of GA or LB medium with shaking at 37°C. Samples were collected and PYO was quantified once every 12 hours. Briefly, a 5-ml volume of culture was mixed with 3 ml of chloroform. After vortexing for 5 min, the sample supernatant was removed and 2 ml of 0.2 M HCl was added to the tube. PYO was extracted in the aqueous pink layer and spectrophotometrically determined at 520 nm [48,49]. Concentrations converted to micrograms of PYO produced per milliliter of culture were measured by multiplying the optical density at 520 nm (OD_520_) by 17.072 [50]. A standard sample of PYO was purchased from Cayman Chemical (Ann Arbor, MI, USA).

### Supplementation of the cultures with exogenous PCA or PYO

To determine whether phenazine feedback affected the expression of the two *phz* operons, the cultures of mutants with the truncated *lacZ* fusions in the chromosome were supplemented with different concentrations of exogenous PCA or PYO during the exponential phases. The PCA sample was generously provided as a gift by Dr. Xu’s research group. PYO was prepared and collected by our laboratory as described by Frank & Demoss [51]. Briefly, one volume of cell-free culture supernatant was added to two volumes of chloroform and shaken for at least 5 min. PYO was extracted from the chloroform into a 0.2 N HCl solution (deep red). When the color changed from red to blue with the addition of NaOH buffer (pH = 10), the blue PYO was again extracted into chloroform. This procedure was repeated 5 times, finally generating PYO powder following the evaporation of the chloroform. High concentration PCA and PYO were dissolved in ethanol; the same volume of ethanol was supplied to the cultures as the negative control. During the following cultivation, the samples were collected at fixed intervals and β-galactosidase-specific activities were analyzed.

### β-Galactosidase assay

All bacterial strains were grown with shaking at 200 rpm in 500-ml conical flasks containing 150 ml LB or GA medium at 37°C. Samples of strain PAO1 and its derivatives were collected after a specified periods of growth. β-Galactosidase-specific activities were determined according to the method of Miller using SDS- and chloroform-treated cells in appropriate amounts [35,52].

### Statistical analysis

All data were analyzed with one-way analysis of variance using the statistical software package SPSS (Chicago, IL, USA).

## Results

### Both *phz1* and *phz2* operons contribute to phenazine production in culture condition

To quantitatively evaluate the specific contribution of the two *phz* loci to phenazine compound production, the two single-deletion mutants (Δ*phz1* and Δ*phz2*) were cultivated in GA or LB medium. The wild-type strain PAO1 was used as the positive control and the double-deletion mutant (Δ*phz1phz2*) as the negative control. Bacterial growth was determined at optical density 600 nm (OD_600_) at 12 hour intervals.

**Fig 2:**
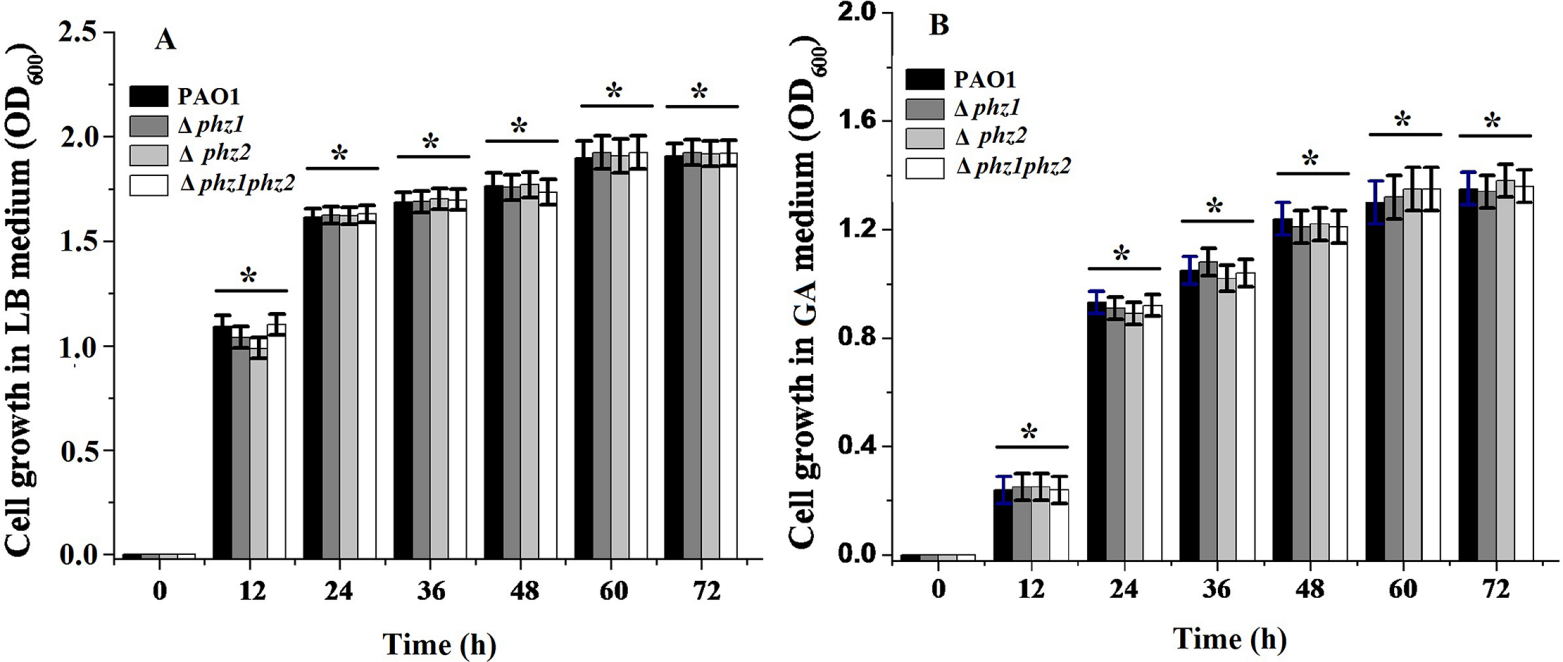
Bacterial growth curves of *P*. *aeruginosa* PAO1 and its derivatives in LB and GA medium. Each of the wild-type strain PAO1 and its derivatives was respectively inoculated in 150 ml of LB medium (A) or GA medium (B). Optical density 600 nm was determined at 12 hour intervals. All experiments were performed in triplicate, and each value was presented as the average ± standard deviation. * indicates *P* > 0.05, two-tailed paired Student *t* test.

**Fig 3:**
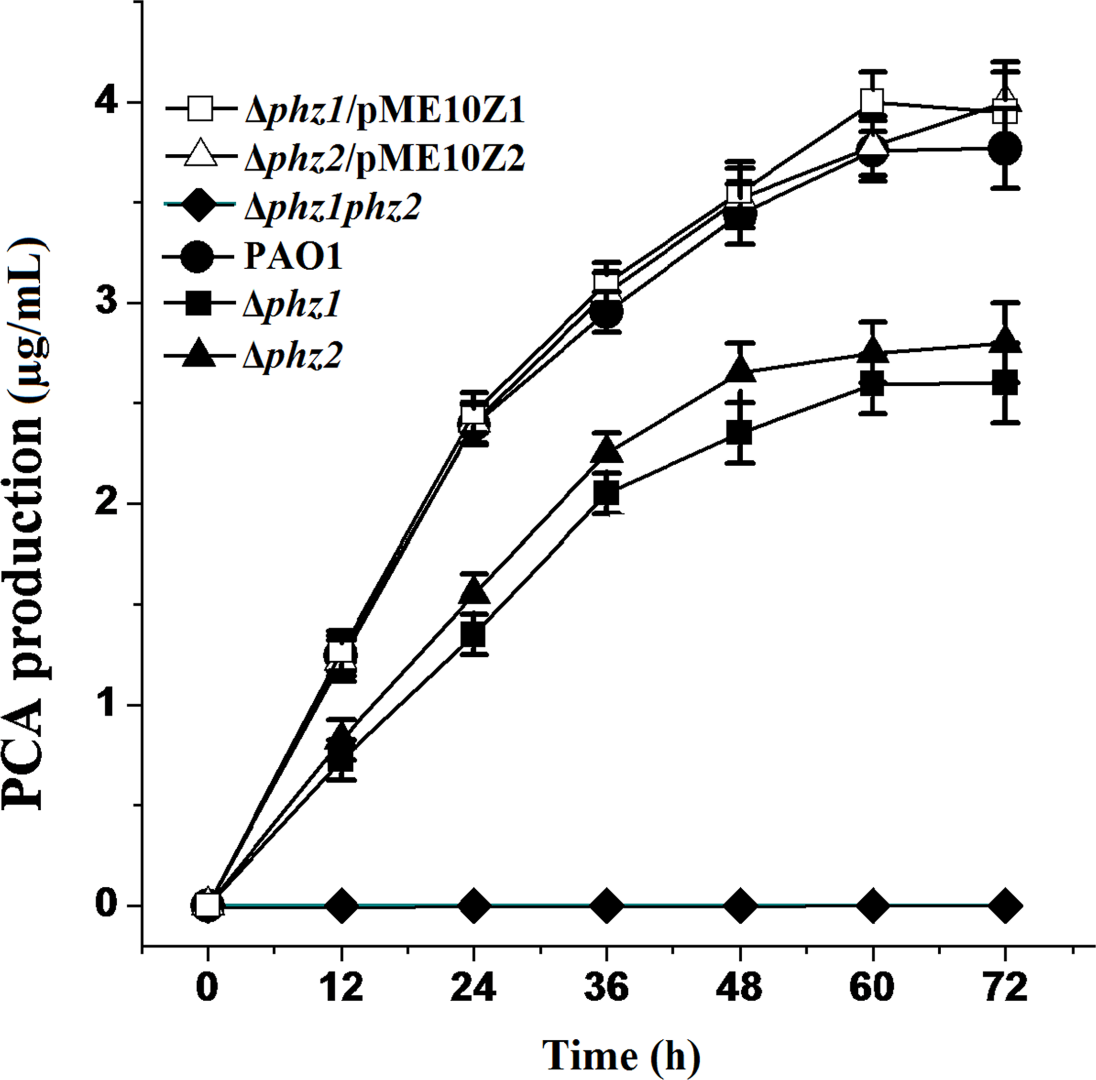
PCA produced by *P*. *aeruginosa* PAO1 and its derivatives in LB medium. All strains including the wild-type strain PAO1 (solid circle), the single-deletion mutant Δ*phz1* (solid square) and Δ*phz2* (solid triangle), the double-deletion mutant Δ*phz1phz2* (solid diamond), the Δ*phz1* mutant complemented with pME10Z1 (open square) and the Δ*phz2* mutant harboring pME10Z2 (open triangle) were grown in LB broth. All experiments were performed in triplicate, and each value was presented as the average ± standard deviation.

**Fig 4:**
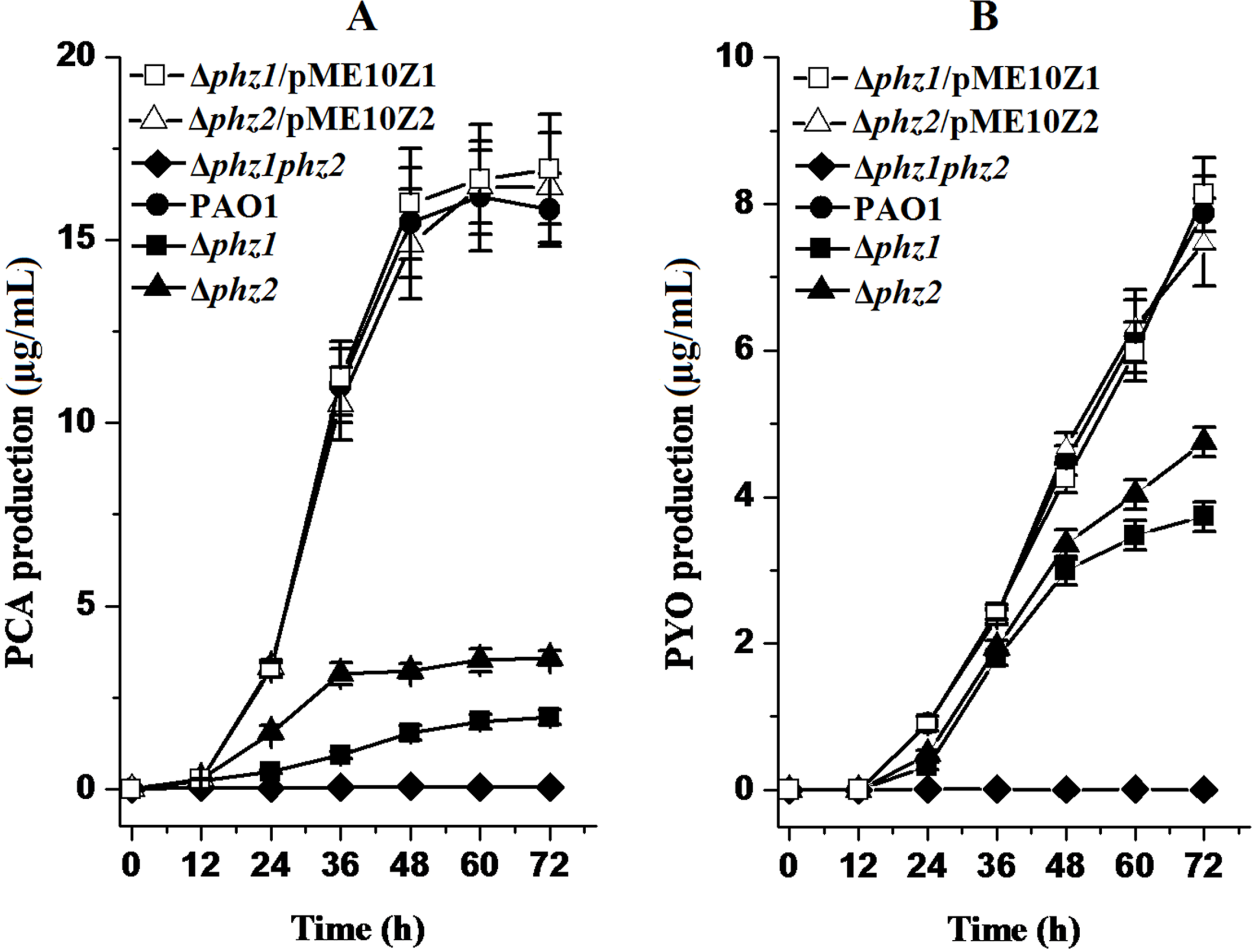
PCA and PYO produced by *P*. *aeruginosa* PAO1 and its derivatives in GA medium. PCA (A) and PYO (B) were biosynthesized by the wild-type strain PAO1 (solid circle) and its derivatives, the single-deletion mutant Δ*phz1* (solid square) and Δ*phz2* (solid triangle), the double-deletion mutant Δ*phz1phz2* (solid diamond), the Δ*phz1* mutant harboring pME10Z1 (open square) and the Δ*phz2* mutant containing pME10Z2 (open triangle) in GA medium. All experiments were performed in triplicate, and each value was presented as the average ± standard deviation.

Although the bacterial growth of the pseudomonad strains differed from one another in different media, there were no significant differences in the growth curves in GA or LB medium between the wild-type strain PAO1 and its derivatives (Fig 2). Thus, the deletion of the two *phz* loci exerted no effects on bacterial growth. As shown in Fig 3, PCA production was decreased in the single-deletion mutant Δ*phz1* and Δ*phz2* compared to the wild-type strain PAO1. However, the amount of PYO produced respectively by the mutant Δ*phz1*, Δ*phz2* and the parental strain PAO1 were same and negligible in LB medium following spectrophotometric analysis, suggesting that LB medium was not suitable for PYO biosynthesis. As shown in Fig 4, PCA and PYO produced in the single-deletion mutant Δ*phz1* and Δ*phz2* in the GA medium were lower than those obtained in the wild-type strain PAO1. No matter which medium (LB or GA medium) was used to culture them, the single-deletion mutant Δ*phz1* and Δ*phz2* produced less amounts of PCA and PYO than the wild-type strain PAO1. Moreover, the Δ*phz2* mutant did not produce much more PCA and PYO compared with the Δ*phz1* mutant, suggesting that the two *phz* operons contributed equally to phenazine production.

To further confirm the contribution of *phz1* and *phz2* operons to PCA and PYO production, complementation experiments were performed by expression of the *phz1* and *phz2* on a shuttle vector. We found that PCA and PYO produced in the Δ*phz1* and Δ*phz2* mutants harboring the pME6010 plasmid were equal to those produced in the Δ*phz1* and Δ*phz2* mutants without the pME6010 plasmid. As shown in Figs 3 and 4, When pME10Z1 harboring the whole *phz1* operon or pME10Z2 bearing the whole *phz2* operon were introduced into the Δ*phz1* or Δ*phz2* mutants, respectively, PCA and PYO production were restored to the level produced by the wild-type strain PAO1.

### Total expression levels of *phz2* and *phz1* operon are cross-upregulated in the absence of *phz1* and *phz2* operon respectively

**Fig 5:**
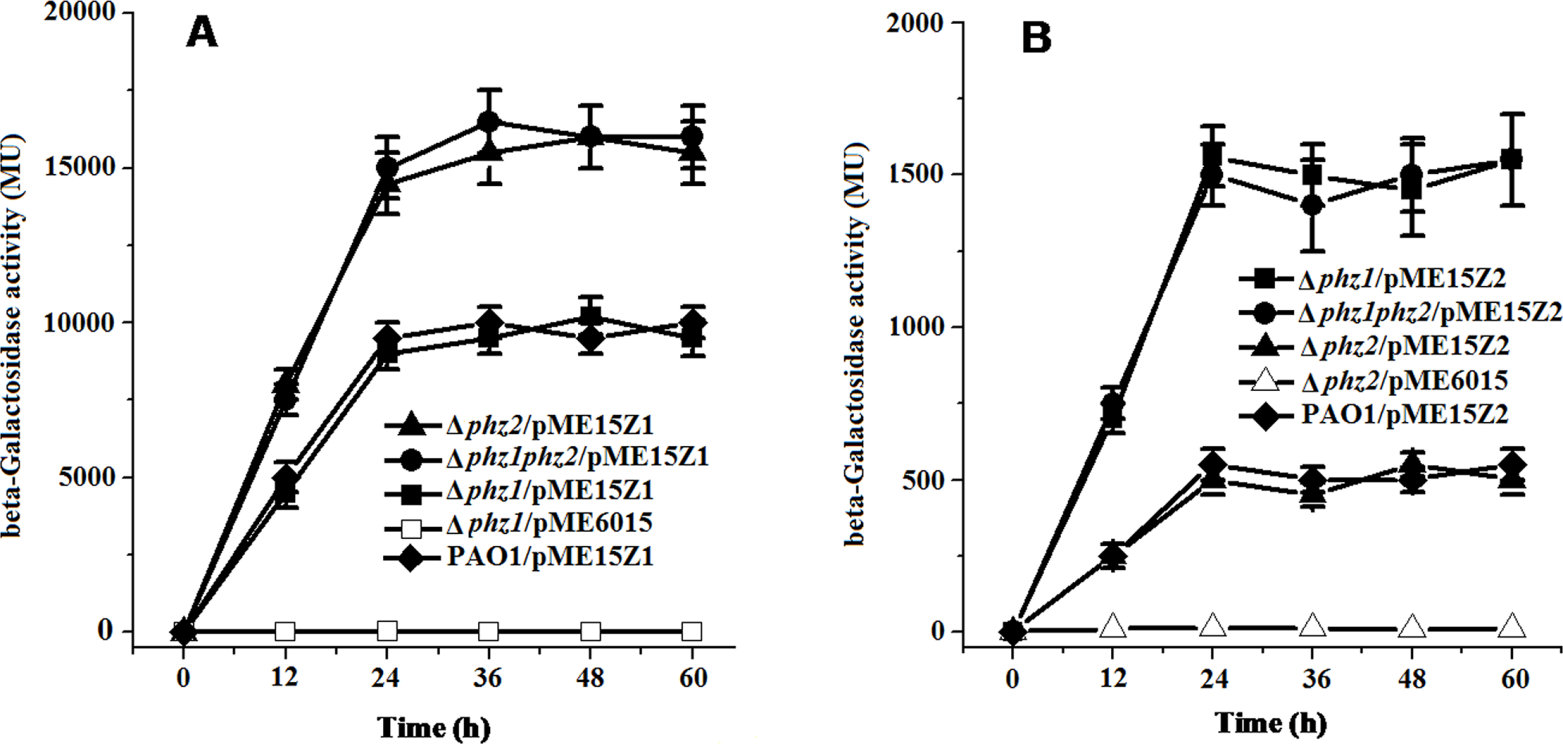
Translational fusion constructs pME15Z1 (*phz1′-′lacZ*) and pME15Z2 ((*phz2′-′lacZ*) generated to study regulation between two phenazine-producing loci. **(**A) β-Galactosidase activities were produced by pME15Z1 in the double-deletion mutant Δ*phz1phz2* (solid circle), the single-deletion mutant Δ*phz1* (solid square), Δ*phz2* (solid triangle), and in the wild-type PAO1 (solid diamond). pME6015 in mutant Δ*phz1* (open square) served as the negative control. (B) β-Galactosidase activities were produced by pME15Z2 in the Δ*phz1phz2* mutant (solid circle), the Δ*phz1* mutant (solid square), the Δ*phz2* mutant (solid triangle), and the wild-type PAO1 (solid diamond). pME6015 in the single-deletion mutant Δ*phz2* (open triangle) served as the negative control. All experiments were performed in triplicate, and each value was presented as the average ± standard deviation.

To explore whether *phz2* exerts any regulatory effects on the expression of the *phz1* operon, the translational fusion construct pME15Z1 (*phzA1'-'lacZ*) was transferred into the single-deletion mutant Δ*phz1*, Δ*phz2* or the double-deletion mutant Δ*phz1phz2*. We found that the β-galactosidase activity of the *phzA1'-'lacZ* fusion construct in the Δ*phz2* or Δ*phz1phz2* mutants was enhanced by 50% compared to the Δ*phz1* mutant (Fig 5A). These results suggested that deletion of the *phz2* operon led to increased expression of the *phz1* operon.

To determine whether the *phz1* exerts any influences on the expression of the *phz2* locus, the translational fusion construct pME15Z2 (*phzA2'-'lacZ*) was delivered into the single-deletion mutants Δ*phz1*, Δ*phz2* or the double-deletion mutant Δ*phz1phz2*. We found that the β-galactosidase activity of the *phzA2'-'lacZ* fusion construct in the double-deletion mutant Δ*phz1phz2* or the single-deletion mutant Δ*phz1* was enhanced 3-fold compared to the single-deletion mutant Δ*phz2* (Fig 5B). These results indicated that deletion of the *phz1* operon led to enhancement of *phz2* operon expression.

**Fig 6:**
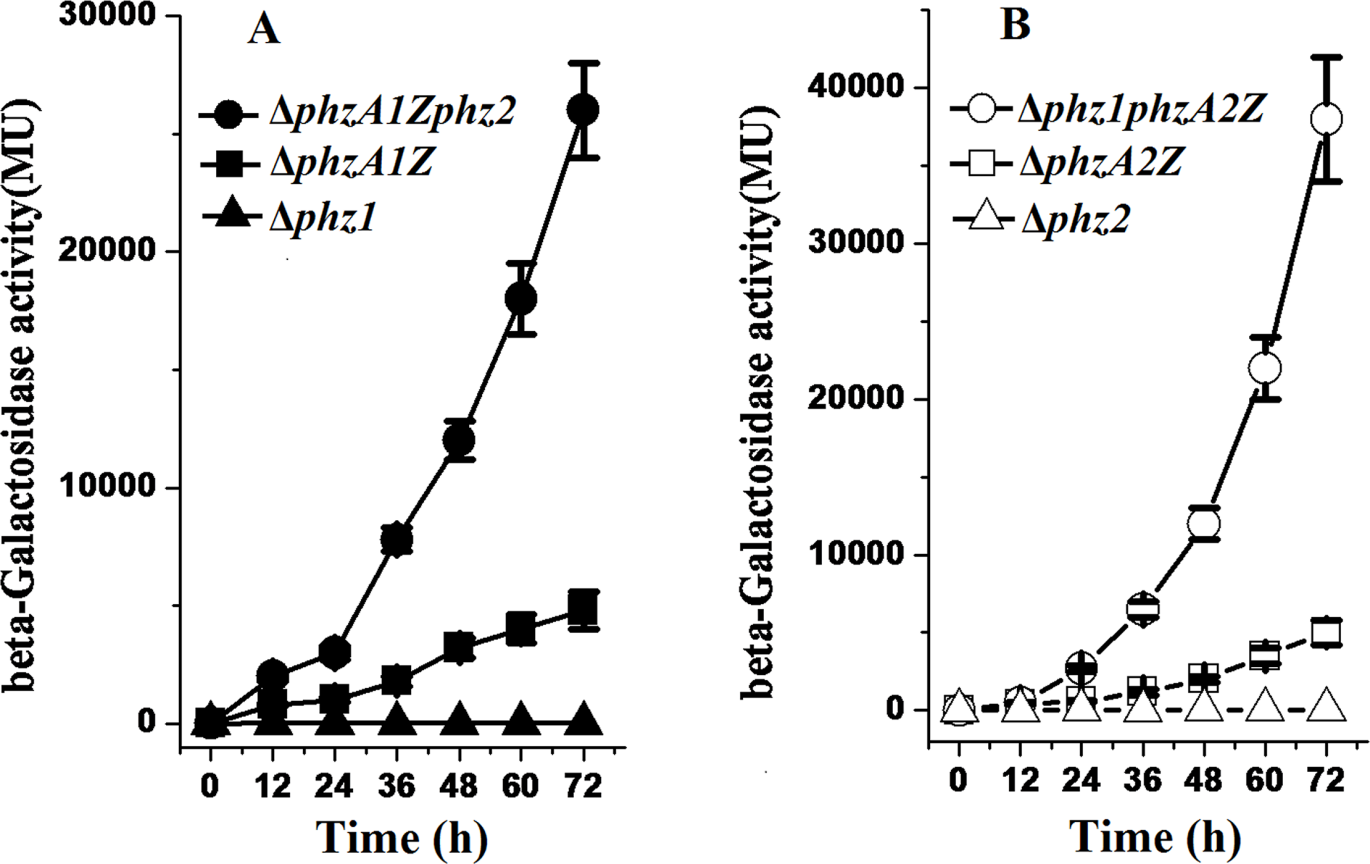
Enhancement of expression of one *phz* operon in the absence of the other operon. (A) Expression of the translational fusion *phz1-lacZ* in chromosome in the presence of the *phz2* operon (in the mutant Δ*phzA1Z*, solid squares) or in the absence of the *phz2* operon (in the mutant Δ*phzA1Zphz2*, solid circles). (B) Expression of the translational fusion *phz2-lacZ* in chromosome in the presence of the *phz1* operon (in the mutant Δ*phzA2Z*, open squares) or in the absence of the *phz1* operon (in the mutant Δ*phz1phzA2Z*, open circles). β-Galactosidase activities determined in the single-deletion mutant Δ*phz1* (solid triangles) or Δ*phz2* (open triangles) were used as the negative controls. Each point was the mean of three measurements ± standard deviation.

To truly and precisely reflect the expression of the two *phz* operons under natural conditions and to eliminate the negative effects due to copies of the translation fusion plasmid in the deletion mutants, a set of fusion mutants in which *phzA1* or *phzA2* was fused in frame on the chromosome with a truncated *lacZ* reporter were constructed using the wild-type strain PAO1, the single deletion mutant Δ*phz1* or Δ*phz2* as receptor strains. The nearly identical growth curves of the translational fusion mutants Δ*phzA1Z*, Δ*phzA1Zphz2*, Δ*phzA2Z*, *and* Δ*phz1phzA2Z* grown in LB or GA broth indicated that their growth rates were not affected by the mutation or fusion in the two *phz* loci regions (data available on figshare). As shown in Fig 6A, the expression of the translational fusion construct *phzA1-lacZ* on the chromosome in the Δ*phzA1Zphz2* mutant was enhanced 2- to 4- fold compared to the Δ*phzA1Z* mutant. This result was consistent with the result of the translational fusion expressed from the plasmid discussed above, suggesting that the expression of the *phz1* operon was up-regulated in the absence of the *phz2* operon. As shown in Fig 6B, the expression of the translaitonal fusion construct *phz2-lacZ* on the chromosome in the Δ*phz1phzA2Z* mutant was elevated 6 folds compared to the Δ*phzA2Z* mutant. This result was similar to the result obtained in the translational fusion on the plasmid, indicating that the expression of the *phz2* operon was up-regulated in the absence of the *phz1* operon.

### The transcription of the *phz2* operon increases in the absence of the *phz1*, the transcription of the *phz1* does not in the absence of the *phz2*

**Fig 7:**
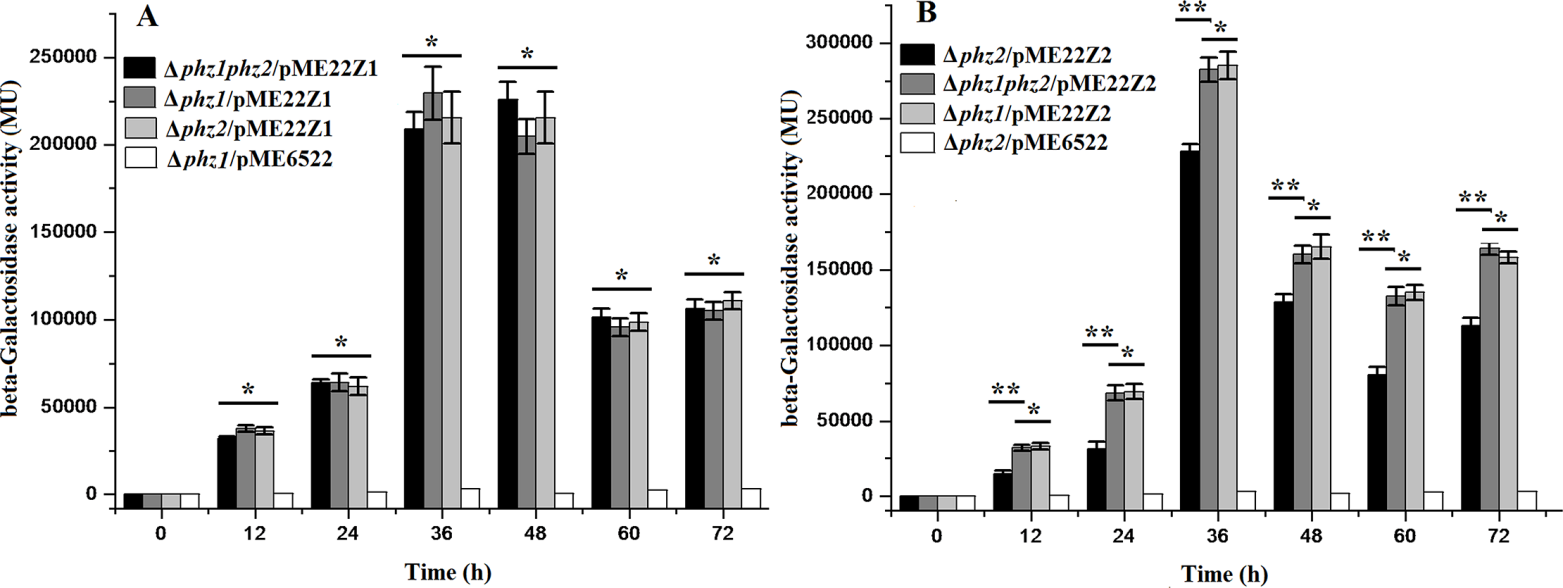
The transcription level assay of one *phz* operon in the absence or in the presence of the other one *phz* operon. (A) β-Galactosidase activities were produced by pME22Z1 in the double-deletion mutant Δ*phz1phz2* (black column), the single-deletion mutant Δ*phz1* (grey column) and mutant Δ*phz2* (light grey column). pME6522 in the mutant Δ*phz1* (white column) served as the negative control. (B) β-Galactosidase activities were produced by pME22Z2 in the mutant Δ*phz1phz2* (grey column), Δ*phz1* (light grey column) and Δ*phz2* (black column). pME6522 in the Δ*phz2* mutant (white column) served as the negative control. All experiments were performed in triplicate, and each value was presented as the average ± standard deviation. *indicates *P* > 0.05, **indicates *P* < 0.01, two-tailed paired Student *t* test.

To determine whether the cross-regulation between the two *phz* operons occurred at the transcriptional or post-transcriptional level, two transcriptional fusion constructs [pME22Z1 (*phz1-lacZ*) and pME22Z2 (*phz2-lacZ*)] were created in pME6522. The β-galactosidase activities of the two transcriptional fusion constructs were measured in the wild-type strain PAO1 and its mutation derivatives. The β-galactosidase activity of pME22Z1 in the double-deletion Δ*phz1phz2* mutant was nearly identical to that in the single-deletion Δ*phz1* mutant (Fig 7A), suggesting that the transcription of the *phz1* operon was not affected by the presence or absence of the *phz2* operon. However, the β-galactosidase activity of pME22Z2 was higher (20 to 30%) in the double-deletion mutant Δ*phz1phz2* than that in the single-deletion mutant Δ*phz2* (Fig 7B), suggesting that the transcription of the *phz2* operon was moderately enhanced by the absence of the *phz1* operon.

### Roles of PCA and PYO in the regulation of *phz* expression

**Fig 8:**
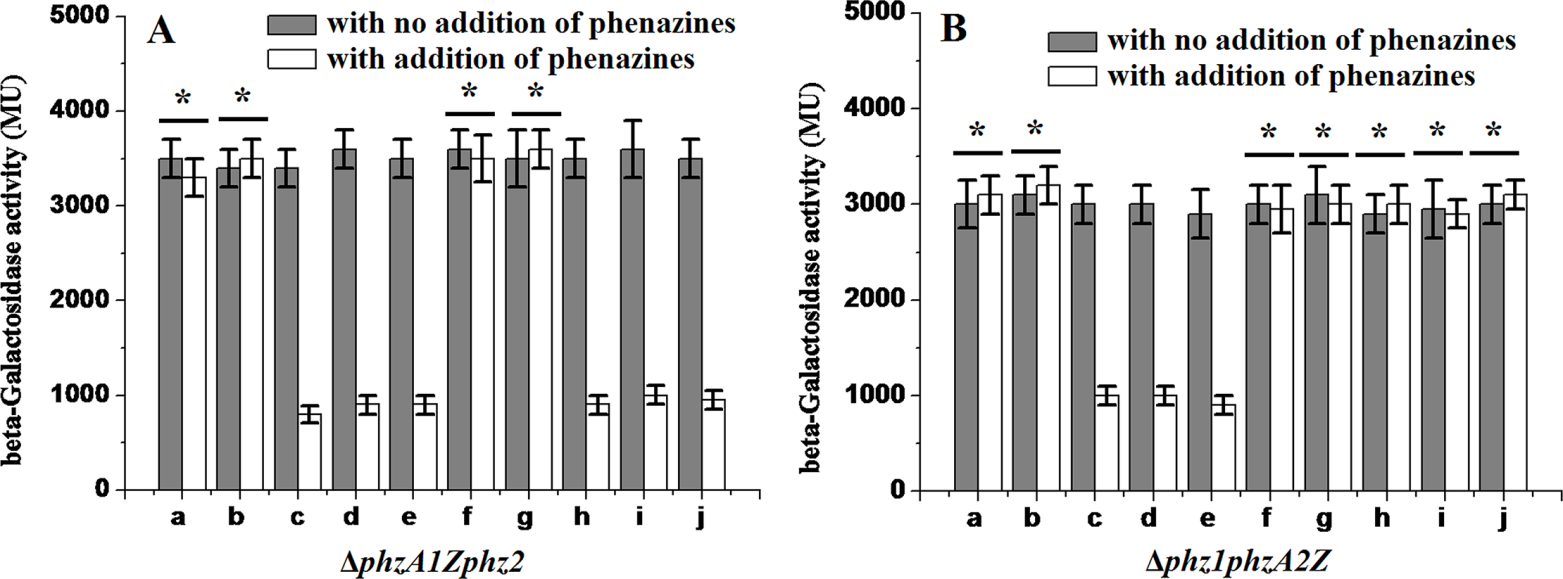
Effects of exogenous phenazines on expression of the *phz* operon. (A) β-Galactosidase activities expressed in the mutant Δ*phzA1Zphz2* in the presence of exogenous PYO or PCA. (B) β-Galactosidase activities expressed in the mutant Δ*phz1phzA2Z* in the presence of exogenous PYO or PCA. a-e, PYO was added to samples with final concentration of 0.08, 0.16, 0.32, 0.64 and 1.28 μg/ml; f-j, PCA was added with 0.025, 0.25, 0.5,1.0, and 2.0 μg/ml. All values were measured after 24 hour of addition. Data reported were the means of triplicate experiments ± standard deviations. * indicates *P* > 0.05, two-tailed paired Student *t* test.

Because PCA and PYO are the main exo-products of the enzymes encoded by the *phz* operons, we tested whether these products have a regulatory effect on the *phz* expression. When a higher concentration of exogenous PYO (>0.32 μg/ml) was added, the β-galactosidase activity in the Δ*phzA1Zphz2* mutant’s culture was reduced compared to that supplemented with ethanol as negative control. These results suggested that PYO accumulation in the culture suppressed the expression of the *phz1* operon. If the concentration of PYO added was low (<0.16 μg/ml), no effect on *phz1* operon expression was observed (Fig 8A). Similar results were obtained in the Δ*phz1phzA2Z* mutant’s culture with the addition of PYO (Fig 8B). The β-galactosidase activity in the Δ*phz1phzA2Z* mutant’s culture was not affected by the addition of exogenous PCA, suggesting that higher concentrations of PCA did not exert negative regulatory effects on the expression of the *phz2* operon. However, expression of β-galactosidase in the Δ*phzA1Zphz2* mutant’s culture was repressed by the addition of exogenous PCA, indicating that the expression of *phz1* was inhibited when high level of PCA accumulated in the culture.

## Discussion

In this study, we constructed a series of *phz* deletion mutants and evaluated the specific contribution of two *phz* operons to phenazine biosynthesis in PAO1. In LB or GA medium, the mutant Δ*phz2* produced slightly more phenazines than the mutant Δ*phz1*. However, in *P*. *aeruginosa* PA14, the *phz1*-deficient mutant produced more PCA than the *phz2*-deleted mutant, suggesting that regulation should be different for the expression of the two *phz* operons in two strains despite the fact that the sequences of *phz* operons and their promoter regions in both strains were extremely identical (>99%) [32]. Our results obtained in two types of media supported the conclusion that the *phz2* operon is active and functional in the wild-type strain PAO1. The function of the *phz2* should not be ignored because it produces phenazines in the LB or GA medium. This conclusion was also supported by the previous work by Mavrodi et al. [16,17]. In their report, PCA was detected in extracts from the transformants when each copy of the two *phz* operons was cloned into an *E*. *coli—P*. *aeruginosa* shuttle vector and then introduced into the non-phenazine-producing strain *P*. *fluorescens* M4-80R or *E*. *coli* JM109. However, their study just verified that the *phz2* was similar to the *phz1* and had the same ability to produce phenazines. In other previous reports, the *phz1* operon was shown to play a major role in producing phenazines because it produced the majority of PYO in the wild-type strain PAO1 in LB medium. Therefore, it was suggested that the *phz2* could not substitute for the *phz1* in the biosynthesis of PYO [33,34]. However, it was possible that the *phzC1* mutant did not produce blue pigment in LB medium in their report because the amount of PYO produced by the *phz2* operon was too low to make the LB medium plate blue, not because the *phz2* produced no PYO. As a matter of fact, the *phz2* operon produced the same amount of PYO in GA medium as the *phz1* during the first 36 hours of cultivation (Fig 4B). Therefore, the blue pigment (PYO) could be biosynthesized by both the *phz1* and *phz2* operons in the parental strain PAO1. This conclusion was also confirmed by the results from our translational fusion *phz1′-′lacZ* and *phz2′-′lacZ* constructed in the pME6015 plasmid in the PAO1 strain.

However, the mechanism by which the two *phz* operons function under natural conditions in the parental strain PAO1 is not clear. To answer this question, we examined the expression of two operons in PAO1 and its derivatives using the *lacZ* reporter gene. We found that the expression of one *phz* operon dramatically increased when the other operon was deleted, suggesting that one *phz* operon could compensate for the absence of the other operon by up-regulating its expression level. We postulated that there would be a homeostatic regulatory mechanism which mediates the expression of the two *phz* operons. To test this hypothesis, we further constructed the translational fusion mutants on the chromosome with the truncated *lacZ* in frame. The assessment of β-galactosidase activities in two pair of mutants (Δ*phzA1Z/*Δ*phzA1Zphz2*, Δ*phzA2Z/*Δ*phz1phzA2Z*) confirmed that a homeostatic balance did exist between the two *phz* operons. Thus, when one *phz* operon (*phz1* or *phz2*) does not function, the other operon would be up-regulated to compensate for the decrease in phenazine production. This similar finding had been reported before in other pseudomonad species. For example, 2,4-diacetylphloroglucinal (DAPG) and pyoluteorin (PLT) display an inverse relationship in *P*. *fluorescens* CHA0 in which each metabolite activates its own biosynthesis while repressing the synthesis of the other metabolite [53,54]. Moreover, phloroglucinol (a precursor of DAPG) is responsible for the inhibition of pyoluteorin production in *P*. *fluorescens* Pf-5 [55]. In *Pseudomonas* sp. M18, one *phz*-deletion mutant M18Z1 produced less PCA, but more pyoluteorin (PLT) [56]. In bio-control strains, homeostatic balance exists during the biosynthesis of secondary metabolites and will compensate for the loss of one antibiotic by overproducing another, thereby maintaining total antibiotic production and bio-control ability [57]. Similarly, the maintenance of the two *phz* operons in *P*. *aeruginosa* PAO1 by the homeostatic balance would keep phenazine production stable, which would be beneficial to its pathogenicity in the host.

In an attempt to gain additional insight into the mechanism for this homeostatic regulation, we created the transcriptional fusion pME22Z1 and pME22Z2 and transformed them into the derivative mutants. β-Galactosidase activities shown that no significant changes occurred at the *phz1* transcription level in the presence or absence of the *phz2*. Combined with the translational fusions’ data, we speculated that the cross-regulation mediating the *phz1* expression occurred at the post-transcriptional level and less likely at the transcriptional level. Interestingly, the transcription of the *phz2* increased moderately in the absence of the *phz1*. Meanwhile, the translational fusion analysis shown that the expression level of the *phz2* increased significantly (more than 3 times) in the absence of the *phz1*. Therefore, we suggested that the cross-regulation between the two *phz* operons might mediate the *phz2* expression at both the transcriptional and post-transcriptional levels.

Based on sequence analysis, the two *phz* loci differed markedly in their upstream regions although they possessed 98.3% identity in their open reading frame regions [31]. These differences may serve as a platform for cross-regulating two *phz* operons and contribute to phenazine biosynthesis. In *P*. *aeruginosa* M18, the 5’ long region in the *phz1* and *phz2* mRNA was demonstrated to post-transcriptionally mediate the expression of two phenazine producing loci [28, 58]. Meanwhile, it was confirmed that RsmA could negatively regulate the *phz1* expression and positively mediate the *phz2* expression at post-transcriptional level [58]. Due to the high identity between strains M18 and PAO1 in their *phz* operons, they may share similar structures or mechanisms involved in the differential mediation of the two phenazine biosynthesis operons. In PCA and PYO feedback assay, exogenous PYO inhibition in PAO1 strain in our study was consistent with the previous work did by Dietrich et al. [27]. However, while exogenous PCA did not exert an effect on the *phz2* expression, it exhibited a negative effect on the *phz1* expression. These results may also provide some clues into the homeostatic interplay between the two *phz* operons. Although we described an initial characterization of the relationship between the two *phz* operons and identified a homeostatic balance between them, we could not explore the precise expression levels of the two *phz* operons in the wild-type strain under natural conditions. This issue should be addressed in future studies.
